# Clinical Outcomes of Early Endoscopic Transpapillary Biliary Drainage for Acute Cholangitis Associated with Disseminated Intravascular Coagulation

**DOI:** 10.3390/jcm10163606

**Published:** 2021-08-16

**Authors:** Akihiro Sekine, Kazunari Nakahara, Junya Sato, Yosuke Michikawa, Keigo Suetani, Ryo Morita, Yosuke Igarashi, Fumio Itoh

**Affiliations:** 1 Department of Gastroenterology and Hepatology, St. Marianna University School of Medicine, Kawasaki 216-8511, Japan; akihiro.sekine@marianna-u.ac.jp (A.S.); j2sato@marianna-u.ac.jp (J.S.); y2michikawa@marianna-u.ac.jp (Y.M.); y2igarashi@marianna-u.ac.jp (Y.I.); fitoh@marianna-u.ac.jp (F.I.); 2 Department of Gastroenterology and Hepatology, Kawasaki Municipal Tama Hospital, Kawasaki 214-8525, Japan; k2suetani@marianna-u.ac.jp; 3 Department of Gastroenterology, Morita Hospital, Sagamihara 252-0159, Japan; r2morita@marianna-u.ac.jp

**Keywords:** acute cholangitis, disseminated intravascular coagulation, endoscopic biliary drainage, endoscopic retrograde cholangiopancreatography, clinical outcome

## Abstract

Acute cholangitis (AC) is often associated with disseminated intravascular coagulation (DIC), and endoscopic transpapillary biliary drainage (EBD) under endoscopic retrograde cholangiopancreatography (ERCP) is a treatment of choice. However, no evidence exists on the outcomes of EBD for AC associated with DIC. Therefore, we retrospectively evaluated the treatment outcomes of early EBD and compared endoscopic biliary stenting (EBS) and endoscopic nasobiliary drainage (ENBD). We included 62 patients who received early EBD (EBS: 30, ENBD: 32) for AC, associated with DIC. The rates of clinical success for AC and DIC resolution at 7 days after EBD were 90.3% and 88.7%, respectively. Mean hospitalization period was 31.7 days, and in-hospital mortality rate was 4.8%. ERCP-related adverse events developed in 3.2% of patients (bleeding in two patients). Comparison between EBS and ENBD groups showed that the ENBD group included patients with more severe cholangitis, and acute physiology and chronic health evaluation II score, systemic inflammatory response syndrome score, and serum bilirubin level were significantly higher in this group. However, no significant difference was observed in clinical outcomes between the two groups; both EBS and ENBD were effective. In conclusion, early EBD is effective and safe for patients with AC associated with DIC.

## 1. Introduction

Acute cholangitis (AC) is often associated with disseminated intravascular coagulation (DIC), which can be fatal without prompt and appropriate treatment intervention. Treatment of the primary disease causing DIC remains the most important factor in the resolution of the pathological conditions underlying DIC, and the prognosis of patients with DIC may be markedly affected by the treatment outcome of the primary disease [1].

Endoscopic transpapillary biliary drainage (EBD) under endoscopic retrograde cholangiopancreatography (ERCP) is the first choice of treatment for AC [2,3]. Endoscopic sphincterotomy (EST) is generally performed before EBD to facilitate insertion of a device into the bile duct or prevention of post-ERCP pancreatitis [4,5]. Moreover, bile outflow can be expected not only through the stent but also through the papilla opened by EST. Nevertheless, when AC is combined with DIC, EBD without EST is generally required because of the high risk for post-EST bleeding. Furthermore, in severe AC associated with DIC, poor drainage or clogging in the stent due to the high viscosity of infected bile and hemobilia associated with contact of the device with the bile duct is a concern in EBD.

EBD methods include endoscopic biliary stenting (EBS) and endoscopic nasobiliary drainage (ENBD). EBS is an internal drainage method with no discomfort and no loss of electrolytes or fluid. In contrast, ENBD is an external drainage method with the advantages of monitoring the bile, performing bile cultures, and washing the catheter. However, patients undergoing ENBD treatment will be uncomfortable because of the transnasal tube and may even pull it out. A few studies compared EBS and ENBD in cases of severe AC. The majority of previous reports demonstrated that no difference existed in the safety and efficacy between EBS and ENBD [6,7,8,9], but a report that ENBD demonstrates better drainage than EBS also exists [10]; nonetheless, no sufficient evidence exists. Furthermore, no study exists on the treatment outcomes of EBD for AC associated with DIC.

Therefore, we conducted this study to evaluate the treatment outcomes of early EBD performed within 24 h after the diagnosis of AC associated with DIC and to further compare the outcomes between EBS and ENBD. This is a single-center, retrospective study. To our knowledge, this study is the first to evaluate the role of EBD in AC associated with DIC.

## 2. Materials and Methods

### 2.1. Patients

In this retrospective study, we investigated the clinical data of 5637 consecutive patients who received ERCP between April 2006 and March 2019 at St. Marianna University School of Medicine Hospital. The inclusion criteria were (1) EBD performed for AC associated with DIC; (2) initial EBD for naïve papilla; (3) EBD performed within 24 h after the diagnosis of AC associated with DIC; and (4) age ≥ 20 years. The exclusion criteria were (1) past history of choledochojejunostomy; (2) history of EST; (3) placement of biliary stent or nasobiliary drainage catheter; (4) EBD performed at >24 h after the diagnosis of AC, and (5) lack of sufficient data in the medical record.

All patients provided written informed consent for the endoscopic procedures. This study was approved by the institutional review board of St. Marianna University School of Medicine (approval number: 5357).

### 2.2. Endoscopic Procedures

ERCP was performed using a duodenoscope with patients under moderate/deep sedation. In general, we performed bile duct cannulation by contrast cannulation or wire-guided cannulation. When biliary cannulation was difficult, we attempted the pancreatic guidewire method or the pancreatic stent placement method. In principle, precut, EST, or stone removal was not performed for patients with DIC. However, the decision to perform precut, EST, or stone removal was left to the discretion of the attending physician. We placed a 7-Fr plastic stent for EBS or a 6-Fr nasodrainage catheter for ENBD. All patients received blood tests 3 h after ERCP.

All ERCP procedures were conducted under the supervision of an expert who performed ≥1000 ERCP procedures (initials: K.N., Y.M., K.S. and R.M.).

### 2.3. Measurements

We retrospectively examined the following parameters: patient backgrounds, details of endoscopic procedures, clinical outcomes including the clinical success rate for AC, DIC resolution rate, mortality rate, and ERCP-related adverse events. Then, we and compared these factors between patients who received EBS (EBS group) and those who received ENBD (ENBD group).

### 2.4. Definitions

The diagnosis and severity of AC were determined according to the Tokyo Guidelines 2018 [11]. The diagnosis of DIC was based on a DIC score of ≥4 according to the DIC diagnostic criteria in Japan [12] (Table 1). Early ERCP was defined as ERCP performed within 24 h after the diagnosis of AC associated with DIC. For bile duct cannulation, the conventional method included contrast cannulation and wire-guided cannulation. The duration of procedure time measured from insertion to removal of the scope by reviewing the nursing records. DIC resolution was defined as a decrease in the DIC score to ≤3 within 7 days after EBD. Clinical success of AC was defined as a reduction in serum bilirubin and inflammation parameters and disappearance of signs of cholangitis such as abdominal pain and fever within 7 days after EBD. The diagnosis and severity of adverse events, including pancreatitis, bleeding, and perforation, were determined according to the consensus guidelines provided by Cotton et al. [13]. Hyperamylasemia was defined as an increase in the serum amylase level that was three-fold or higher than the normal limit (>396 IU/L) without associated abdominal pain after ERCP. The hospitalization period included the time required for endoscopic stone removal in additional ERCP sessions.

### 2.5. Statistical Analysis

Categorical variables were compared using chi-squared test and Fisher’s exact test. Continuous parameters were compared using Student’s *t*-test. *p* values of <0.05 were considered to indicate significance. The statistical analysis was performed using R version 3.4.1 (R Foundation, Vienna, Austria).

## 3. Results

### 3.1. Patient Characteristics

**Figure 1:**
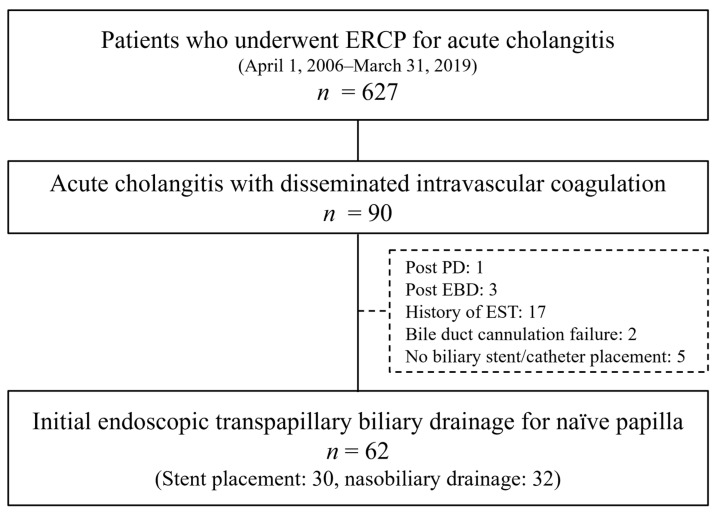
Flowchart of the patient-selection process. ERCP, endoscopic retrograde cholangiopancreatography; PD, pancreatoduodenectomy; EST, endoscopic sphincterotomy; EBD, endoscopic transpapillary drainage.

Among 5637 patients who received ERCP during the study period, 627 patients received ERCP for AC. Of these 627 patients, 90 (14.4%) presented with AC complicated with DIC. Among these 90 patients, 28 were excluded due to the following conditions: post-pancreatoduodenectomy (one patient), post-EBD (three patients), history of EST (17 patients), bile duct cannulation failure (two patients), no stent/catheter placement (five patients). Finally, 62 patients fulfilled the eligibility criteria and were included in the analysis (Figure 1).

Table 2 shows the patient’s characteristics. Their mean age was 78 years, and 65% were men. The predominant cause of AC was bile duct stone (80.6%). AC was found to be severe in 50 (80.6%), moderate in 10 (16.1%), and mild in 2 (3.2%) patients. The mean acute physiology and chronic health evaluation II (APACHE II) score, DIC score, and systemic inflammatory response syndrome (SIRS) score were 13.8, 5.5, and 2.7, respectively. The most used antibiotic was meropenem (62.9%). As an anticoagulant therapy for DIC, recombinant soluble human thrombomodulin and antithrombin were administered in 45 (72.6%) and 35 (56.5%) patients, respectively (there is some overlapping).

### 3.2. Endoscopic Procedures

The details of the endoscopic procedures are presented in Table 3. Selective bile duct cannulation was achieved by the conventional method in 50 patients (80.6%). Only one patient (1.6%) received pre-cut and achieved successful bile duct cannulation. Although procedures for the papilla and stone removal were not performed in most cases, EST and stone removal were performed in seven (11.3%) and four (6.5%) patients, respectively. The patients who underwent EST did not meet the diagnostic criteria of DIC before ERCP due to lack of blood test items, but were diagnosed with DIC by a blood test 3 h after ERCP. EBS and ENBD were performed in 30 (48.4%) and 32 (51.6%) patients, respectively. Pancreatic stent for the prevention of post-ERCP pancreatitis was placed in 13 patients (21.0%). The mean procedure duration for ERCP was 31.4 min.

### 3.3. Clinical Outcomes

**Figure 2:**
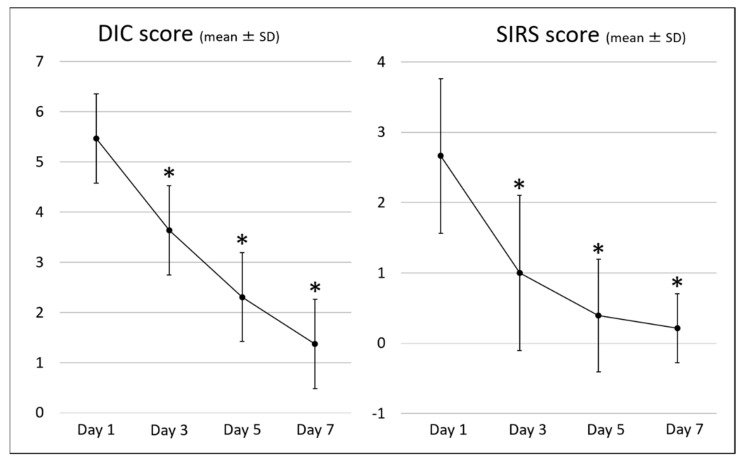
DIC scores and SIRS scores. DIC, disseminated intravascular coagulation; SIRS, systemic inflammatory response syndrome; * *p* < 0.05 vs. baseline.

**Figure 3:**
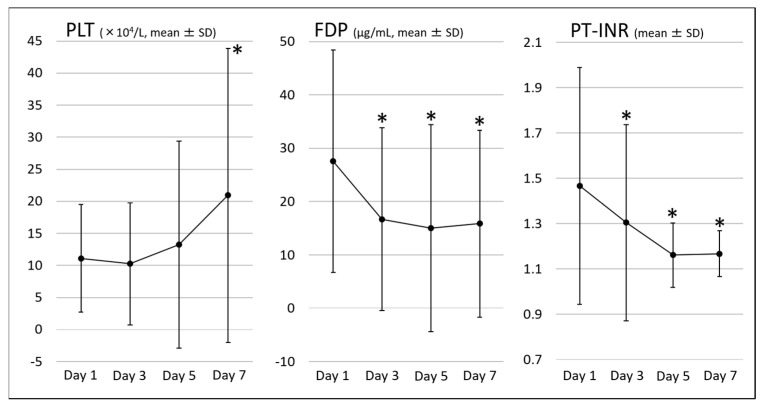
Parameters related to disseminated intravascular coagulation. PLT, platelet; FDP, fibrin degradation product; PT-INR, prothrombin time-international normalized ratio; * *p* < 0.05 vs. baseline.

**Figure 4:**
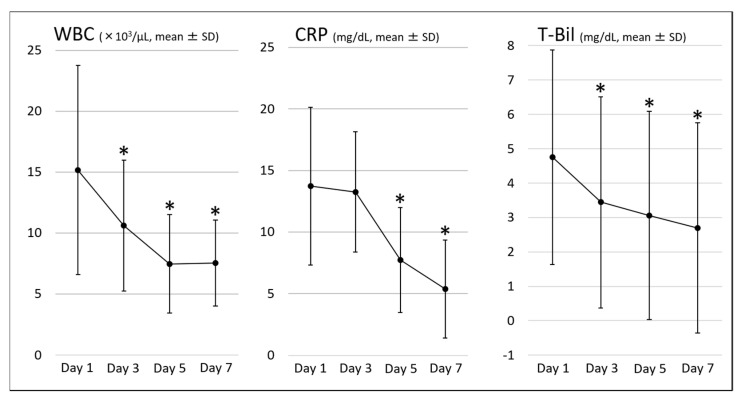
Parameters related to acute cholangitis. WBC, white blood cell; CRP, C-reactive protein; T-Bil, total bilirubin; * *p* < 0.05 vs. baseline.

Table 4 shows the clinical outcomes. The clinical success rate for AC and the DIC resolution rate on day 7 were 90.3% and 88.7%, respectively. Changes in the DIC score and SIRS score, the parameters related to DIC, and the parameters related to AC are shown in Figure 2, Figure 3 and Figure 4, respectively. All these parameters showed significant improvement on day 7 compared with those on day 1 of the diagnosis of AC associated with DIC.

The mean hospitalization period was 31.7 days, and the in-hospital mortality rate was 4.8%. Two patients died due to exacerbation of AC and DIC, and another patient died due to ventilator-associated pneumonia. Of the 50 cases with bile duct stone, 47 cases underwent stone removal during the same hospital stay. In the first ERCP session, four patients in the ENBD group underwent stone removal. After improvement of AC and DIC, 23 patients in the EBS group and 20 patients in the ENBD group underwent stone removal. No patient underwent cholecystectomy during the same hospital stay.

### 3.4. Adverse Events

The adverse events are presented in Table 5. The rate of ERCP-related adverse events was 3.2% (2/62). Post-EST bleeding and Mallory–Weiss bleeding occurred in one patient each during endoscopic procedure. Bleeding could be controlled by endoscopic hemostasis by clipping without blood transfusion in both patients. The patient with EST bleeding received anticoagulant therapy with recombinant thrombomodulin, while the patient with Mallory–Weiss did not receive anticoagulant therapy. There was no rebleeding in both cases. Although hyperamylasemia was observed in six patients (6.4%), no patient developed pancreatitis. Two patients pulled out the ENBD catheter, of which one received additional EBS, and the other received stone removal without stent placement.

### 3.5. Comparison of EBS and ENBD Groups

Comparison between EBS and ENBD groups is presented in Table 6. In the patient backgrounds, the ENBD group contained patients with significantly more severe cholangitis (*p* = 0.02). Moreover, the APACHE II score (*p* < 0.01), the SIRS score (*p* = 0.04), and the total bilirubin level (*p* < 0.01) were significantly higher in the ENBD group. Although no statistically significant difference was found, the DIC score tended to be higher in the ENBD group (5.1 vs. 5.8, *p* = 0.09). These results indicated that ENBD was selected for more critically ill patients with hyperbilirubinemia.

In contrast, although the duration of hospitalization tended to be longer in the ENBD group (27.6 days vs. 35.6 days, *p* = 0.15), no significant difference was found in endoscopic procedures, clinical outcomes, and adverse events between the EBS and ENBD groups.

## 4. Discussion

DIC is a life-threatening condition that necessitates prompt and appropriate treatment. Because controlling the primary disease that caused DIC is the most essential treatment for DIC [1,14,15], EBD is the most important treatment for AC associated with DIC. However, a concern related to EBD for patients with severe AC associated with DIC is poor drainage or clogging in the stent due to the high viscosity of infected bile and hemobilia associated with contact of the device with the bile duct, and no report exists regarding the treatment outcomes of EBD for AC associated with DIC. Therefore, in the present study, we evaluated the treatment outcomes of early EBD for AC associated with DIC. We found that EBD performed within 24 h after the diagnosis of AC associated with DIC is effective and safe, with a clinical success rate for AC of 90.3%, a DIC resolution rate of 88.7%, and an ERCP-related adverse event rate of 3.3%. The DIC score, the parameters related to DIC, and the parameters related to AC were improved within 7 days after EBD. A previous meta-analysis reported that early EBD performed within 24 h from presentation was associated with reduced mortality in patients with AC [16], and our study results also showed that early EBD performed within 24 h is effective for patients with AC associated with DIC.

For patients with AC associated DIC, EBD without EST is generally required because of the high risk for post-EST bleeding. In the present study, seven patients (11.3%) received EST, and among them, one patient (14.3%) developed post-EST bleeding. A meta-analysis conducted by Sawas et al. [17] reported that EBD with EST carries higher risks for post-ERCP bleeding and EBD with and without EST are equally effective drainage methods for severe AC. Theoretically, the concern about performing EBD without EST is the development of post-ERCP pancreatitis from pancreatic duct orifice blockage. However, no case of post-ERCP pancreatitis was found in this study. Moreover, a meta-analysis showed no significant difference in the incidence of post-ERCP pancreatitis [17]. Therefore, EST may not be feasible during an acute phase of AC associated with DIC.

EBD is performed by either EBS or ENBD. A few studies compared EBS and ENBD in cases of severe AC [6,7,8,9,10]. Most previous reports, including randomized controlled trials (RCTs) [6,7], showed that no difference was found between EBS and ENBD in terms of their safety and efficacy in severe AC. However, an RCT conducted by Zang et al. [10] reported an increased rate of blockage in the EBS group and a greater decrease in liver enzyme levels in the ENBD group; nonetheless, no sufficient evidence exists on this subject. Furthermore, for AC associated with DIC, no report that compared EBS and ENBD exists. Therefore, in the present study, we compared the patient backgrounds, the safety and effectiveness between EBS and ENBD in patients with AC associated with DIC, and our results showed that EBS and ENBD were equally effective and safe for this condition. Nevertheless, ENBD was selected for more critically ill patients, such as those with severe cholangitis, high APACHE II score, and high total bilirubin level. ENBD may be more appropriate for such patients because it is an external drainage procedure with advantages of the ability to monitor, aspirate, and wash the bile through the catheter. However, because of patient discomfort due to the transnasal catheter, the possibility of self-extraction of the catheter in ENBD exists, especially in elderly or confused patients. In the present study, two patients with a confused mental state pulled out the nasobiliary catheter and required re-ERCP. Therefore, for confused or elderly patients who cannot tolerate an ENBD, an EBS may be a better option.

Although treating the primary disease of DIC is the most important point when managing infection-related DIC [1], the efficacy of anticoagulant drugs for DIC, such as recombinant human soluble thrombomodulin and antithrombin, has been reported [18,19,20,21,22,23]. In this study, treatment outcomes may be affected by anticoagulant therapy. However, only a few reports on anticoagulant therapy for AC-induced DIC exist [14,15,24,25], so very little evidence exists that can form a basis for the selection of anticoagulant agents for this condition.

Several limitations were present in this study. An accurate comparison of the drainage ability between EBS and ENBD was not possible because the patient backgrounds were different between the two groups, i.e., more critically ill patients belonged to the ENBD group. A selection may have caused bias in the endoscopic procedures, such as EST, stone removal, and the method of EBD, because the endoscopic procedures were left to the discretion of the attending endoscopist. Treatment outcomes may be affected by anticoagulant therapy and antibiotics [14,15,24,25]. Furthermore, this study used a retrospective design, and the number of patients was small because of the rare nature of the subject disease, thereby indicating that a large-scale, prospective study is necessary to confirm our findings. However, to our knowledge, this study is the first to evaluate the role of EBD in AC associated with DIC, and we believe that this study contains useful information.

In conclusion, early EBD performed within 24 h after the diagnosis of AC associated with DIC is effective and safe for treating patients with AC associated with DIC.

## Figures and Tables

**Table 1:** Diagnostic criteria for disseminated intravascular coagulation as defined by the Japanese Association for Acute Medicine.

Diagnostic Criteria for Disseminated Intravascular Coagulation	Points
Systemic inflammatory response syndrome (SIRS) criteria *	
>3	1
0–2	0
Platelet count (PLT), ×103/L	
<80 or >50% decrease within 24 h	3
>80 and <120; or >30% decrease within 24 h	1
>120	0
Prothrombin time international-normalized ratio (PT-INR)	
>1.2	1
<1.2	0
Fibrin/fibrinogen degradation products (FDPs), μg/L	
>25	3
>10 and <25	1
<10	0
Diagnosis of disseminated intravascular coagulation ≥4 points	
* Systemic inflammatory response syndrome (SIRS) criteria ▪ Fever > 38 °C or < 36 °C ▪ Heart rate > 90 beats per minute ▪ Respiratory rate > 20 breaths per minute or a PaCO_2_ < 32 mmHg ▪ White blood cell count > 12,000/µL or < 4000/µL or > 10% bands	

**Table 2:** Patient backgrounds.

	*n* = 62
Age (mean ± SD)	77.7 ± 9.4
Sex (Male/Female)	40/22
Etiology of acute cholangitis	
Bile duct stone/Malignant biliary stricture	50/12
Severity of acute cholangitis	
Mild/Moderate/Severe	2/10/50
APACHE II score (mean ± SD)	13.8 ± 6.4
DIC score (mean ± SD)	5.5 ± 1.3
SIRS score (mean ± SD)	2.7 ± 1.1
Serum parameters (mean ± SD)	
WBC (×10^3^/μL)	15.2 ± 8.6
CRP (mg/dL)	13.7 ± 6.4
T-bil (mg/dL)	4.8 ± 3.1
AST (IU/L)	312.2 ± 393.6
ALT (IU/L)	252.4 ± 264.3
Plt (×10^4^/L)	11.1 ± 8.4
FDP (μg/mL)	27.6 ± 20.9
PT-INR	1.47 ± 0.52
Antibiotics	
Carbapenem	46
Sulbactam/Cefoperazone	8
Tazobactam/Piperacillin	3
Others	5
Anticoagulant drugs	
Thrombomodulin	45
Antithrombin	35
Gabexate	39
Gamma globulin	42

SD, standard deviation; APACHE, acute physiology and chronic health evaluation; DIC, disseminated intravascular coagulation; SIRS, systemic inflammatory response syndrome.

**Table 3:** Endoscopic procedures.

	*n* = 62
Bile duct cannulation	
Conventional method	50
Pancreatic guidewire method	10
Pancreatic stent placement method	1
Precut	1
Procedure for papilla	
EST	7
Incision range (small/moderate)	3/4
EPBD	2
Biliary drainage	
EBS	30
ENBD	32
Stone removal	4
Use of mechanical lithotripsy	0
Incidental pancreatography	29
Prophylactic pancreatic stenting	13
Procedure time (min, mean ± SD)	31.4 ± 19.8

EST, endoscopic sphincterotomy; EPBD, endoscopic papillary balloon dilation; EBS, endoscopic biliary stenting; ENBD, endoscopic nasobiliary drainage; SD, standard deviation.

**Table 4:** Clinical outcomes.

	*n* = 62
Clinical success rate for acute cholangitis (% (n))	90.3 (56)
DIC resolution rate (% (n))	88.7 (55)
Number of ERCP sessions (mean ± SD)	2.0 ± 0.6
Hospitalization period (day, mean ± SD)	31.7 ± 21.4
Mortality rate (% (n))	4.8 (3)

DIC, disseminated intravascular coagulation; ERCP, endoscopic retrograde cholangiopancreatography; SD, standard deviation.

**Table 5:** Adverse events.

	*n* = 62
Total ERCP-related adverse events (% (n))	3.2 (2)
Pancreatitis (% (n))	0 (0)
Bleeding (% (n))	3.2 (2)
Perforation (% (n))	0 (0)
Stent dysfunction (% (n))	3.2 (2)
Hyperamylasemia (% (n))	6.4 (4)

ERCP, endoscopic retrograde cholangiopancreatography.

**Table 6:** Comparison between EBS and ENBD groups.

	EBS Group (*n* = 30)	ENBD Group (*n* = 32)	*p*-Value
Patient backgrounds			
Age (mean ± SD)	79.2 ± 8.7	76.5 ± 9.9	0.27
Etiology of acute cholangitis			
Bile duct stone/Malignant disease	24/6	26/6	1.00
Severity of acute cholangitis			
Mild/Moderate/Severe	2/8/20	0/2/30	0.02
APACHE II score (mean ± SD)	11.4 ± 3.9	16.2 ± 7.4	<0.01
DIC score (mean ± SD)	5.1 ± 0.9	5.8 ± 1.3	0.09
SIRS score (mean ± SD)	2.3 ± 1.0	2.9 ± 1.1	0.04
Serum parameters (mean ± SD)			
WBC (×10^3^/μL)	15.0 ± 8.9	15.3 ± 8.6	0.90
CRP (mg/dL)	12.7 ± 6.9	14.7 ± 5.8	0.23
T-bil (mg/dL)	3.4 ± 1.7	6.0 ± 3.6	<0.01
AST (IU/L)	344.0 ± 515.6	290.6 ± 240.5	0.61
ALT (IU/L)	268.3 ± 306.6	243.5 ± 224.8	0.72
Plt (×10^4^/L)	11.2 ± 5.7	11.0 ± 10.4	0.90
FDP (μg/mL)	25.1 ± 15.7	29.9 ± 24.9	0.41
PT-INR	1.50 ± 0.71	1.43 ± 0.26	0.62
Antibiotics			
Carbapenem	22	24	0.89
Sulbactam/Cefoperazone	4	4	0.78
Tazobactam/Piperacillin	2	1	0.95
Others	2	3	0.94
Endoscopic procedures			
Conventional cannulation	24	27	0.33
EST	4	3	1.00
Stone removal	0	4	0.11
Procedure time (min, mean ± SD)	32.5 ± 20.0	30.38 ± 19.89	0.67
Clinical outcomes			
Clinical success for acute cholangitis (% (n))	96.7 (29)	84.4 (27)	0.36
DIC resolution rate (% (n))	93.3 (28)	84.4 (27)	0.67
Number of ERCP sessions (mean ± SD)	2.0 ± 0.61	2.0 ± 0.57	0.83
Hospitalization period (mean ± SD)	27.6 ± 11.8	35.6 ± 26.9	0.15
Mortality rate (% (n))	0 (0)	9.4 (3)	0.24
Adverse events			
Bleeding (% (n))	3.3 (1)	3.1 (1)	1.00
Stent dysfunction (% (n))	0 (0)	6.3 (2)	0.49

EBS, endoscopic biliary stenting; ENBD, endoscopic nasobiliary drainage; SD, standard deviation; APACHE, acute physiology and chronic health evaluation; DIC, disseminated intravascular coagulation; SIRS, systemic inflammatory response syndrome; EST, endoscopic sphincterotomy; ERCP, endoscopic retrograde cholangiopancreatography.

## Data Availability

The data presented in this study are available in the article.
